# Consecutive ruptures on a complex conjugate fault system during the 2018 Gulf of Alaska earthquake

**DOI:** 10.1038/s41598-021-85522-w

**Published:** 2021-03-16

**Authors:** Shinji Yamashita, Yuji Yagi, Ryo Okuwaki, Kousuke Shimizu, Ryoichiro Agata, Yukitoshi Fukahata

**Affiliations:** 1 Graduate School of Life and Environmental Sciences, University of Tsukuba, Tsukuba, Ibaraki 305-8572 Japan; 2 Faculty of Life and Environmental Sciences, University of Tsukuba, Tsukuba, Ibaraki 305-8572 Japan; 3 Mountain Science Center, University of Tsukuba, Ibaraki, 305-8572 Japan; 4 COMET, School of Earth and Environment, University of Leeds, Leeds, LS2 9JT UK; 5 Japan Agency for Marine-Earth Science and Technology, 3173-25 Showa-machi, Kanazawa-ku, Yokohama 236-0001 Japan; 6 Disaster Prevention Research Institute, Kyoto University, Uji, Kyoto 611-0011 Japan

**Keywords:** Geophysics, Seismology, Tectonics

## Abstract

We developed a flexible finite-fault inversion method for teleseismic *P* waveforms to obtain a detailed rupture process of a complex multiple-fault earthquake. We estimate the distribution of potency-rate density tensors on an assumed model plane to clarify rupture evolution processes, including variations of fault geometry. We applied our method to the 23 January 2018 Gulf of Alaska earthquake by representing slip on a projected horizontal model plane at a depth of 33.6 km to fit the distribution of aftershocks occurring within one week of the mainshock. The obtained source model, which successfully explained the complex teleseismic *P* waveforms, shows that the 2018 earthquake ruptured a conjugate system of N-S and E-W faults. The spatiotemporal rupture evolution indicates irregular rupture behavior involving a multiple-shock sequence, which is likely associated with discontinuities in the fault geometry that originated from E-W sea-floor fracture zones and N-S plate-bending faults.

## Introduction

**Figure 1:**
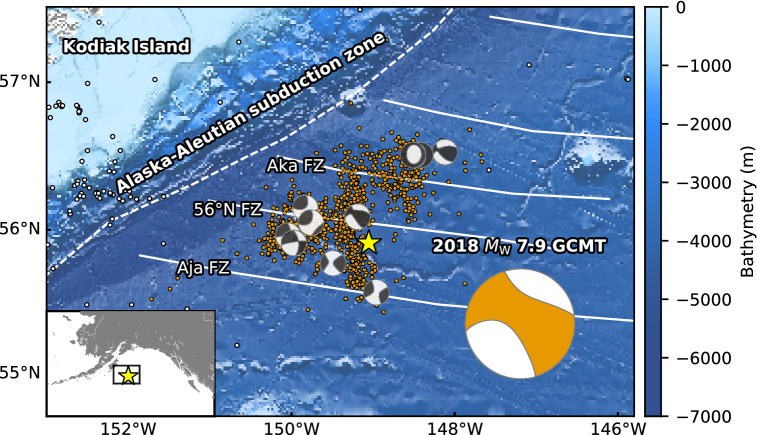
Overview of the source region of the 2018 Gulf of Alaska earthquake. The star is the mainshock epicenter, orange dots are aftershocks (*M* ≥ 3) that occurred within one week of the mainshock, and white dots show background seismicity before the mainshock (*M* ≥ 3.5, 1 January 2008 to 22 January 2018); all epicentral locations are from AEC^1^. The ‘beachball’ diagrams show the GCMT solutions for the mainshock (large, bottom right) and aftershocks with *M* ≥ 3.5. White dashed lines represent plate boundaries^18^, and white solid lines represent fracture zones^19,20^. The background bathymetry is derived from the GEBCO 2020 Grid^21^. The inset map shows the regional setting. This figure was made with matplotlib (v3.1.1)^48^, ObsPy (v1.1.0)^49^, and Generic Mapping Tools (v5.4.5)^50^.

The 23 January 2018 Gulf of Alaska earthquake (moment-magnitude *M*_W_ 7.9^1^) struck offshore Kodiak Island (55.9097°N, 149.0521°W, 10.4 km depth; Alaska Earthquake Center, AEC^1^), in the seaward-region of the Alaska-Aleutian subduction zone. The Global Centroid Moment Tensor (GCMT) project^2, 3^ reported that the 2018 Gulf of Alaska earthquake had strike-slip faulting with a large non-double-couple component (47%). Aftershock seismicity determined by the AEC^1^ shows a lineation extending about 120 km N-S near the epicenter and two aftershock clusters centered about 60 km northeast and about 50 km west from the epicenter (Fig. 1). The GCMT solutions of aftershocks are dominated by strike-slip faulting, but include normal and reverse faulting (Fig. 1).

Several pioneering studies that built finite-fault models based on the aftershock distribution demonstrated that the 2018 Gulf of Alaska earthquake ruptured a quasi-orthogonal multiple-fault system oriented approximately N-S and E-W^4–8^. However, it is difficult to adopt a reasonable fault model because the fault model parametrization, number of fault segments, and fault geometries differ by study, partly due to the spatial spread of the aftershock distribution (Fig. 1). Based on the static slip distribution estimated from Global Navigation Satellite System and tsunami data, major slips occurred on E-W-striking segments^5,7,8^. Finite-fault inversions estimated that the maximum slip occurred around the boundary between the crust and uppermost mantle in the N-S-oriented segment^4,6^, which would have played a significant role in tsunami generation. However, it remains challenging to adequately explain the complex characteristics of the observed teleseismic body waveforms by conventional finite-fault inversion methods due to the uncertainty on the fault geometry, which lead to significant model errors.

In the framework of finite-fault waveform inversion, uncertainties on the Green’s function and fault geometry have been the major sources of model errors^9–13^. Those due to uncertainty on the Green’s function arose from a discrepancy between the true and calculated Green’s functions. To mitigate the effect of this uncertainty, Yagi and Fukahata^13^ explicitly introduced the error term of the Green’s function into the data covariance matrix. As a result, their inversion framework allowed the stable estimation of the spatiotemporal distribution of slip-rate, usually without the non-negative slip-rate constraint, which had been commonly applied in conventional waveform inversion methods to obtain a plausible solution^14,15^.

Model errors due to uncertainty on the fault geometry arose from inappropriate assumptions about the fault geometry^11,12^. For strike-slip earthquakes, many seismic stations are distributed in the vicinity of nodal planes where the radiation pattern is sensitive to the assumed fault geometry. An obtained solution can easily be distorted by inappropriate assumptions of strike and dip^12^. These effects can be mitigated by increasing the degrees of freedom in the assumed seismic source model. Shimizu et al.^12^ proposed an inversion method to express slip vectors on the assumed model plane as the seismic potency tensor. Because their method adopts a linear combination of five basis double-couple components^16^, the slip direction is not restricted to the two slip components compatible with the fault direction. Of course, the true fault geometry should be compatible with the actual slip direction. Whilst the teleseismic *P*-wave Green’s function is insensitive to slight changes in the absolute source location, it is sensitive to the assumed focal mechanisms^12,16,17^, and their inversion method enabled the spatiotemporal resolution of not only the detailed rupture evolution, but also variation of the focal mechanism, including information on the fault geometry, which may differ from the assumed model plane.

In this study, we developed a flexible finite-fault inversion framework that can estimate both the rupture evolution and focal mechanism of earthquakes that ruptured along multiple complex fault segments. This method incorporates appropriate smoothness constraints and a high-degree-of-freedom planar model into the inversion framework of Shimizu et al.^12^. Application of our framework to the 2018 Gulf of Alaska earthquake shows that our source model sufficiently reproduced the observed complex waveforms without assumptions on fault geometry. The model also clarified multiple, distinct rupture events in the conjugate fault system that have not been revealed by conventional finite-fault inversion methods.

## Method

In the inversion framework of Shimizu et al.^12^, the seismic waveform $u_{j}$ observed at a station $j$ is given by

1 $$u_{j} \left( t \right) = \mathop \sum \limits_{q = 1}^{5} \mathop \int \limits_{S}^{{}} \left( {G_{qj} \left( {t,{\varvec{\xi}}} \right) + \delta G_{qj} \left( {t,{\varvec{\xi}}} \right)} \right)*\dot{D}_{q} \left( {t,{\varvec{\xi}}} \right)d{\varvec{\xi}} + e_{bj} \left( t \right),$$

where $G_{qj}$ is the calculated Green’s function of the $q$ th basis double-couple component, $\delta G_{qj}$ is the model error on $G_{qj}$ ^13^, $\dot{D}_{q}$ is the $q$ th potency-rate density function on the assumed model plane $S$, $e_{bj}$ is background and instrumental noise, ${\varvec{\xi}}$ represents a position on $S$, and $*$ denotes the convolution operator in the time domain.

Shimizu et al.^12^ represented the assumed model plane $S$ as a rectangle horizontally covering the seismic source region. However, for earthquakes with complex fault geometries, such as the 2018 Gulf of Alaska earthquake, such a horizontal rectangular model plane includes areas beyond the seismic source region. Therefore, we further extended their inversion framework such that a horizontal non-rectangular model plane can be set according to the shape of the ruptured region as estimated from other information (e.g., aftershock seismicity). In other words, we introduced a priori information about the possible ruptured area into the inversion framework. In numerical tests, the use of a non-rectangular model plane improved spatial resolution and computation costs compared to a rectangular one (see Supplementary Material S1 and Figs. S1–S4).

In general, inversions are stabilized by adding smoothness constraints either implicitly or explicitly^22–24^. In the formulation of Shimizu et al.^12^, the smoothness constraints on each potency-rate density function $\dot{D}_{q}$ in space and time are represented as

2 $$\begin{array}{*{20}c} {\nabla^{2} \dot{D}_{q} \left( {t,\xi } \right) + {\upalpha }_{q} = 0,} \\ \end{array}$$

3 $$\begin{array}{*{20}c} {\frac{{\partial^{2} }}{{\partial t^{2} }}\dot{D}_{q} \left( {t,\xi } \right) + {\upbeta }_{q} = 0,} \\ \end{array}$$

where ${\upalpha }_{q}$ and ${\upbeta }_{q}$ are assumed to be Gaussian noise with zero mean and covariances of $\sigma^{2} {\mathbf{\rm I}}$ and $\tau^{2} {\mathbf{\rm I}}$, respectively, where ${\mathbf{\rm I}}$ is an $M \times M$ ($M$ is the number of model parameters) unit matrix. Because they introduced identical Gaussian distributions for all basis components and determined the optimal values of the hyperparameters $\sigma^{2}$ and $\tau^{2}$ by Akaike’s Bayesian information criterion^23,25^, the potency-rate density functions of basis components with relatively high amplitudes become smoother than those of basis components with relatively low amplitudes, which may bias the solution. Thus, when the amplitudes of the potency-rate density functions differ for each basis component, the standard deviations of the smoothness constraints should depend on the amplitude of each basis component.

In this study, we set the standard deviation of the smoothness constraints for each basis double-couple component to be proportional to its amplitude. That is, instead of ${\upalpha }_{q}$ and ${\upbeta }_{q}$, we directly introduced Gaussian noise with zero mean and covariances $\sigma_{q}^{2} {\mathbf{\rm I}}$ and $\tau_{q}^{2} {\mathbf{\rm I}}$, respectively, as

4 $$\begin{array}{*{20}c} {\sigma_{q}^{2} {\rm I} = k^{2} m_{q}^{2} \sigma^{2} {\rm I},} \\ \end{array}$$

5 $$\begin{array}{*{20}c} {\tau_{q}^{2} {\rm I} = k^{2} m_{q}^{2} \tau^{2} {\rm I},} \\ \end{array}$$

where $k$ is a scaling factor and $m_{q}$ is the total potency of the $q$ th basis double-couple component, which is independently derived from the moment tensor solution. To avoid extremely small standard deviations destabilizing the solution, we adjusted $k\left| {m_{q} } \right|$ so that it does not fall below 10% of its maximum absolute value. Following Yagi and Fukahata^13^, we determined the hyperparameters $\sigma^{2}$ and $\tau^{2}$ by Akaike’s Bayesian information criterion^23,25^. In numerical tests, these improved smoothness constraints mitigated the excessive smoothing of the dominant basis component imposed by conventional smoothness constraints and, when combined with a non-rectangular model plane, outperformed the conventional framework (see Supplementary Material S1, Figs. S1–S4 and Table S1).

### Data and fault parameterization

**Figure 2:**
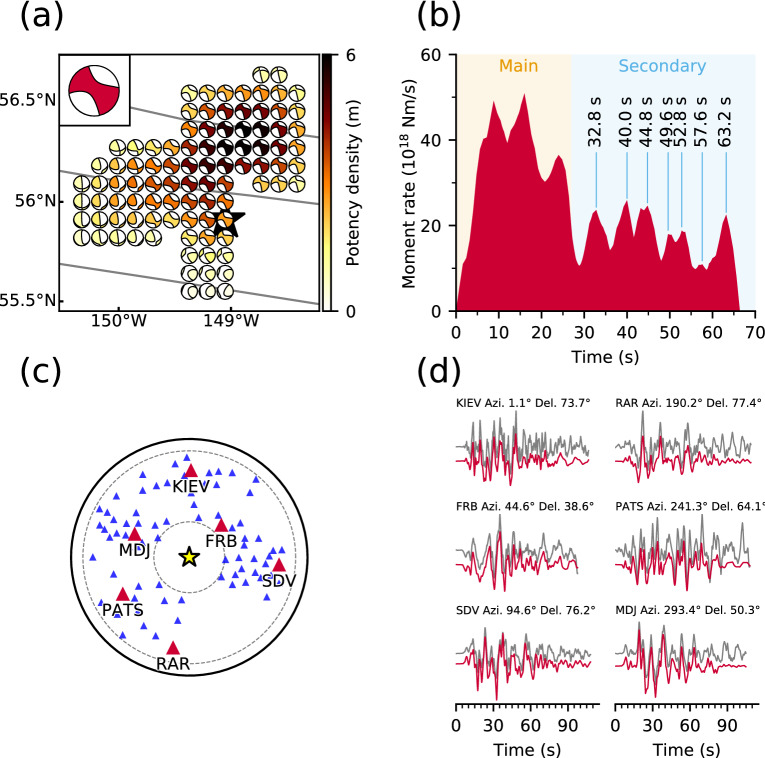
Model setting and summary of results. (**a**) Map projection of the potency density tensor distribution on the assumed model plane. The star and solid lines indicate the epicenter^1^ and fracture zones^19,20^, respectively. Inset is the total moment tensor. (**b**) The moment rate function is divided into the main and secondary rupture stages at 27 s. The individual peaks during the secondary stage correspond to snapshots in Fig. 3b. (**c**) Azimuthal equidistant projection of the station distribution used in the inversion. The star denotes the epicenter, and triangles denote station locations (waveforms for red stations are shown in (**d**)). The inner and outer dotted lines show epicentral distances of 30° and 90°, respectively. (**d**) Comparison of observed waveforms (gray) with synthetic waveforms (red) at the selected stations in (**c**). Each panel is labeled with the station name, azimuth (Azi.), and epicentral distance (Del.) from the mainshock. Waveform comparisons for all stations are shown in Supplementary Fig. S11. This figure was made with matplotlib (v3.1.1)^48^ and ObsPy (v1.1.0)^49^.

We used teleseismic *P* waveforms (vertical components) recorded at stations with epicentral distances of 30–90° (downloaded from the Incorporated Research Institutions for Seismology Data Management Center). Of these, we selected 78 stations, ensuring a high signal-to-noise ratio and an azimuthal coverage^26^ (Fig. 2c), and converted the *P* waveforms to velocity waveforms at a sampling rate of 0.8 s. The theoretical Green’s functions for teleseismic body waves were calculated by the method of Kikuchi and Kanamori^16^ at a sampling rate of 0.1 s, and the attenuation time constraint $t^{*}$ for the *P* wave was taken to be 1.0 s. We adopted a 1-D velocity structure derived from the CRUST1.0 model^27^ (see Supplementary Table S2) to calculate the theoretical Green’s functions. Following Shimizu et al.^12^, we did not low-pass filter the observed waveforms or calculated Green’s functions. For the smoothness constraints, we calculated $m_{q}$ based on the GCMT solution of the 2018 Gulf of Alaska earthquake. The GCMT solution shows that the M1 (strike-slip) component^16^ is more prominent than the others (see Supplementary Table S3), including the M4 (dip-slip) component^16^ (see Supplementary Fig. S4). The scaling factor $k$ in eqs. (4) and (5) was set such that $min\left( {k\left| {m_{q} } \right|} \right) = 1$ (Table S3).

Based on the aftershock distribution, the 2018 Gulf of Alaska earthquake is considered to have occurred on a quasi-orthogonal multiple-fault system^4–8^. To cover the high point density area of aftershocks within one week of the event^1^ (Fig. 2a), we set up a non-rectangular horizontal model plane with a maximum width and length of 130 km, which was expanded using a bilinear B-spline with a knot spacing of 10 km. We adopted the epicenter as that determined by the AEC^1^: 55.9097°N, 149.0521°W. The depth of the model fault plane was set at 33.6 km according to the GCMT centroid depth. For the inversion analysis, we adopted a potency-rate density function on each knot, each representing a linear combination of B-splines at an interval of 0.8 s. The maximum rupture-front velocity, which defines the rupture starting time at each knot, was set to 7.0 km/s to account for the possibility of supershear rupture propagation. The rupture ending time at each knot was set to 65 s from the origin time based on previous inversion results^4,6^. We evaluated the sensitivity of our model by perturbing the model parameters, and the robustness of the new method (see Supplementary Material S2, and Figs. S5, S6 and S9).

## Results

We estimated the spatiotemporal distribution of the potency density tensor for the 2018 Gulf of Alaska earthquake by applying our flexible finite-fault inversion method to teleseismic *P* waveforms. The estimated total moment tensor, calculated by taking the spatial and temporal integrals of the potency-rate density functions, expresses strike-slip faulting, including 36% non-double-couple components (Fig. 2a). The spatial distribution of the potency density tensor, obtained by temporally integrating the potency-rate density functions at each knot, is also dominated by strike-slip focal mechanisms, with a maximum slip of 6 m about 50 km north of the epicenter (Fig. 2a). The moment rate function is elevated over two time periods, separated at 27 s from the origin time: the first period is characterized by three large spikes and the second by numerous smaller spikes (Fig. 2b). The total seismic moment is 14.9 × 10^20^ N m (*M*_W_ 8.05). The synthetic waveforms from the obtained source model well reproduce the observed waveforms (see Supplementary Fig. S11), including those at stations near the nodal planes (Fig. 2d).

**Figure 3:**
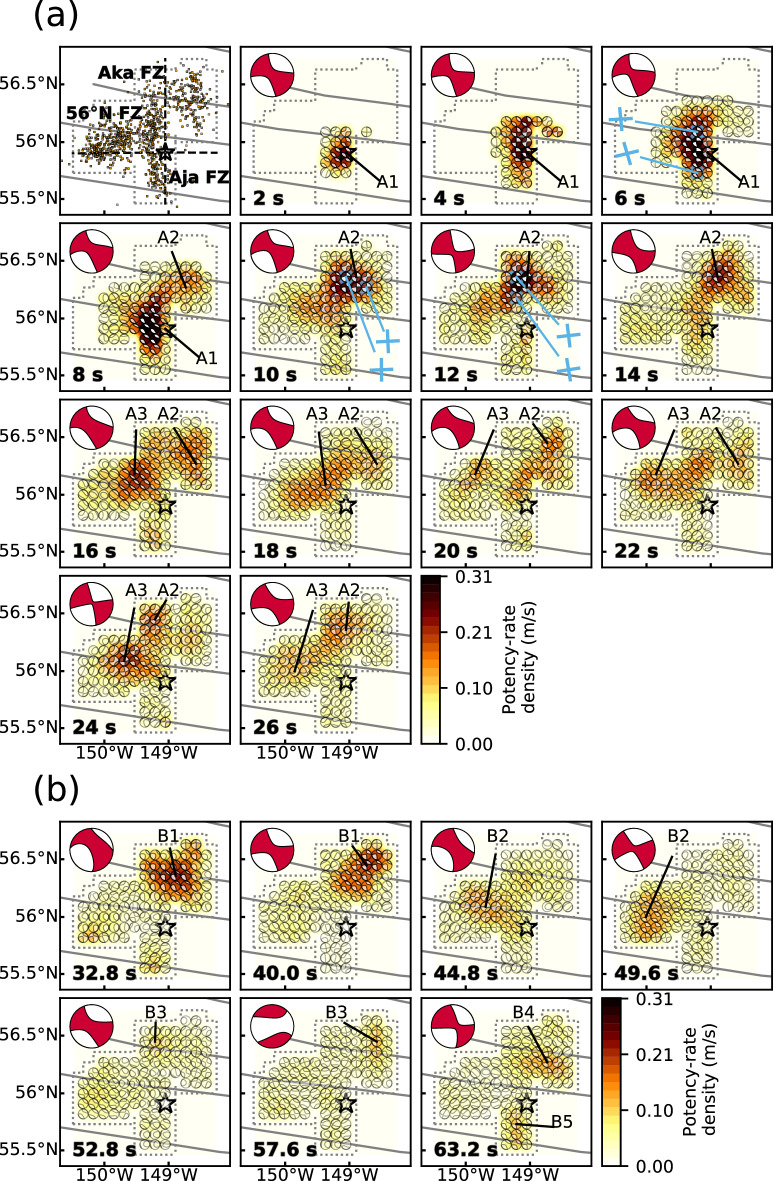
Snapshots of the potency-rate density tensors for (**a**) the main rupture stage A and (**b**) the secondary rupture stage B. The corresponding time after onset for each snapshot is noted at the bottom-left of each panel. The dotted line shows the border of the assumed model plane. The star and solid lines indicate the epicenter^1^ and fracture zones^19,20^, respectively. Blue crosses show the strike directions of small beachball diagrams derived from the potency-rate density tensor. The top-left panel in (**a**) is the epicentral distribution of aftershocks (*M* ≥ 3) that occurred within one week of the mainshock^1^. The large beachball in each panel indicates the corresponding total moment tensor at each time. The dashed lines on the left-top panel of (**a**) are the projection lines used for Fig. 4. This figure was made with matplotlib (v3.1.1)^48^ and ObsPy (v1.1.0)^49^.

**Figure 4:**
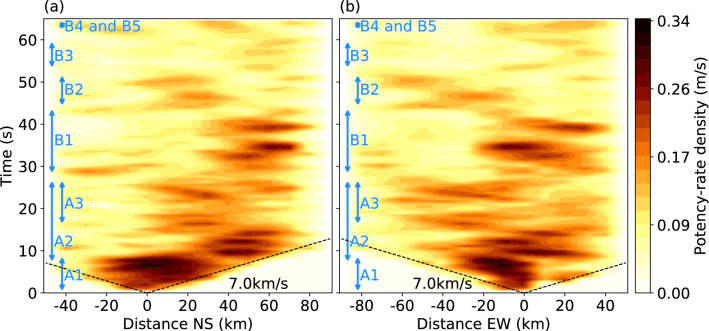
Time evolution of potency-rate density distribution, projected along (**a**) north–south and (**b**) east–west directions, where the positive distance directs toward (**a**) north and (**b**) east from the epicenter. North–south and east–west distances are measured along the dashed lines on the left-top panel of Fig. 3a. The dashed line represents the reference rupture speed. Each rupture phase is annotated on left of each panel. This figure was made with matplotlib (v3.1.1)^48^.

Based on the moment rate function and snapshots of the potency-rate density tensors (Figs. 2b and S12, respectively), we report the detailed rupture history by dividing it into main (A, 0–27 s) and secondary rupture stages (B, 27–65 s). Based on the location, timing, and continuity of the rupture, we further identified three phases (A1–A3) during the main stage and five (B1–B5) during the secondary stage (Figs. 3 and 4).

### Main rupture stage (A)

The initial phase, A1 (0–9 s), started at the hypocenter and propagated bilaterally northward and southward with strike-slip focal mechanisms (snapshot at 2 s in Fig. 3a). Although it is generally difficult to identify the preferred fault plane from the two possible nodal planes in this earthquake, the direction of rupture propagation during phase A1 coincided with the N-S directed nodal plane. The spatial distribution of focal mechanisms shows that the strike of the fault plane gradually rotated counterclockwise from north to south of the epicenter; we obtained a strike/dip of 174°/82° around 20 km north of the epicenter, but 163°/76° around 20 km south of the epicenter (6 s in Fig. 3a). The northward rupture seems to have stagnated near the 56°N fracture zone^28^ (FZ) after about 9 s (Fig. 4a).

Phase A2 (7–27 s) started about 50 km northeast of the epicenter at around 7 s after the origin time and propagated west along the Aka FZ^28^ (8 s in Fig. 3a). This rupture direction is consistent with the obtained E-W strike directions (e.g., 10 s in Fig. 3a). The westward rupture propagated to 149.2°W, where the Aka FZ intersects the N-S aftershock lineation, until 11 s, then turned southward, indicating that the N-S strike direction is the preferred fault plane (12 s in Fig. 3a). The southward rupture halted at around 12 s at the same location where the northward rupture of phase A1 had stagnated at about 9 s (Fig. 4a). After 12 s, a discontinuous rupture occurred along the Aka FZ: ruptures propagating southward and northward from the Aka FZ near 148.6°W are detected at around 16 and 20 s, respectively (Fig. 3a). The rupture on the Aka FZ near 149.2°W is again apparent at around 24 s, and gradually ceased by 27 s.

Phase A3 (16–27 s), started about 40 km northwest of the epicenter, near the 56°N FZ, around 16 s after the origin time (Fig. 3a). This rupture propagated bilaterally to the northeast and southwest until around 18 s, then gradually abated until around 20 s. At that time, another western rupture occurred at the northwest end of the model region and propagated to the south (20 s in Fig. 3a), stagnating at the 56°N FZ about 50 km west of the epicenter at around 22 s (24 s in Fig. 3a).

### Secondary rupture stage (B)

We identified seven peaks in the moment rate function during the secondary rupture stage (Fig. 2b), which we attribute to five phases in the snapshots (Fig. 3b). Phase B1 (28–44 s) occurred along the Aka FZ. In particular, phase B1 ruptures at around 32.8 and 40.0 s were relatively large, and appear as individual peaks in the moment rate function (Figs. 2b and 3b). Phase B2 (44–52 s) mainly ruptured the region west of the epicenter. The rupture at around 44.8 s occurred along the 56°N FZ and that at around 49.6 s struck about 30 km south of the 56°N FZ (Fig. 3b). Phase B3 (53–60 s) occurred mainly northeast of the epicenter, but also struck the intersection of the Aka FZ and the N-S aftershock lineation at around 52.8 s (Fig. 3b). A northward rupture from the Aka FZ was also detected at around 57.6 s. The last peak of the moment rate function corresponds to two independent phases that occurred at around 63.2 s: B4 (62–65 s) ruptured about 20 km south of the Aka FZ and B5 (62–64 s) ruptured about 30 km south of the epicenter (Fig. 3b).

## Discussion

Our inversion results indicate that the main rupture stage (0–27 s after origin) affected segments oriented both N-S and E-W, suggesting that the 2018 Gulf of Alaska earthquake ruptured a conjugate fault system, as proposed in previous studies^4–8^. Our source model suggests that the rupture occurred along weak zones in the sea floor: fracture zones extending E-W and plate-bending faults parallel to N-S magnetic lineaments^29,30^. The N-S plate bending faults have been interpreted as pre-existing oceanic spreading features that were reactivated by subduction of the Pacific Plate^30^. Krabbenhoeft et al.^28^ associated these pre-existing features with the radiation of high-frequency waves based on back-projection and the aftershock distribution (see Supplementary Fig. S13).

A notable irregular rupture propagation highlighted by our inversion results is the northward rupture at around 9 s in phase A1 and the southward rupture at around 12 s in phase A2, both of which stopped near the 56° N FZ (8 and 12 s, respectively, in Figs. 3a and 4a). The N-S aftershock lineation is divided into northern and southern clusters across the 56°N FZ (Fig. 3a). Given the phase A1 and A2 ruptures and the geometrical offset of the N-S aftershock lineation, the northern and southern fault system crossing the 56°N FZ can be regarded as a strike-slip step over. Based on our obtained focal mechanisms, these two N-S faults are both right-lateral strike-slip faults that dip steeply to the west (8 and 12 s in Fig. 3a), and the counterclockwise rotation of the strike angle during phase A1 is consistent with the southern N-S aftershock lineation (6 s in Fig. 3a). Because irregular rupture behaviors are generally a result of geometric complexities, including barriers caused by discontinuous fault steps^31–33^, we interpret that this fault step over caused the rupture to stagnate at around 9 and 12 s.

Multiple sub-events occurring in a conjugate strike-slip fault system have been reported in previous studies^34–38^. In this study, we have shown a causal link between the multiple rupture episodes during the 2018 Gulf of Alaska earthquake (stages A and B) and pre-existing bathymetric features by resolving both the rupture evolution and variation of fault geometry using only teleseismic body waves. Similar observations were made during the *M*_W_ 8.6 2012 Sumatra earthquake in the Wharton basin. That earthquake involved multiple *M*_W_ > 8 sub-events along a conjugate fault system^37,39^, which developed by deep ductile shear localization beneath the brittle upper lithosphere of the oceanic plate^40^.

We evaluated how the newly developed method improved the source model of the 2018 Gulf of Alaska earthquake by performing the inversion analysis with the conventional smoothness constraints^12^ (Fig. S7). The inversion result with the conventional smoothness constraints show general agreement with that obtained by the improved smoothness constraints (Fig. S7). However, the spatiotemporal rupture propagation of the conventional smoothness constraints is smoother than that of the improved ones by the excessive smoothing for the most dominant $M1$ component for the earthquake (Fig. S8), which provides the blurrier image, making it difficult to clearly resolve the multiple sub-events (Figs. 3 and S7).

It is possible that the complex waveforms observed during the 2018 Gulf of Alaska earthquake were contaminated by reverberations due to the bathymetric setting that cannot be reproduced by the theoretical Green’s function, resulting in dummy multiple events^41–44^. We evaluated this possibility by using empirical Green’s functions^45,46^ and confirm that it is unlikely that the multiple rupture stages originated from such reverberations (see Supplementary Material S3 and Fig. S10).

The sub-events that occurred after the main A1 phase can be regarded as early aftershocks missing from global catalogs^47^. Although it is difficult to distinguish whether such early near- to intermediate-field aftershocks were dynamically or statically triggered^47^, it is noteworthy that the rupture propagated from A1 to A2 at more than 5 km/s (see Supplementary Material S2 and Fig. S6); this is faster than the surface wave velocity (3–4 km/s), suggesting that the A2 rupture was triggered by the A1 rupture.

## Conclusions

We developed a finite-fault inversion method for teleseismic *P* waveforms with improved smoothness constraints to obtain source processes for earthquakes with complex multiple-fault ruptures. We applied our inversion method to the 2018 Gulf of Alaska earthquake and estimated its spatiotemporal rupture process. Although the observed waveforms are very complicated, reflecting the complex rupture process and fault geometry, the waveforms calculated from our source model fit well. The obtained source model suggests a complex multiple-shock sequence on a conjugate fault system, consistent with pre-existing bathymetric features. Irregular rupture stagnation about 20 km north of the epicenter may have been promoted by a fault step across a sea-floor fracture zone.

## Supplementary Information

Supplementary information.

## Data Availability

Waveform data was downloaded through the IRIS Wilber 3 system (https://ds.iris.edu/wilber3/find_stations/10607586). Teleseismic waveforms were obtained from the following networks: the Canadian National Seismograph Network (CN; https://doi.org/10.7914/SN/CN); the Caribbean USGS Network (CU; https://doi.org/10.7914/SN/CU); the GEOSCOPE (G; https://doi.org/10.18715/GEOSCOPE.G); the Hong Kong Seismograph Network (HK; https://www.fdsn.org/networks/detail/HK/); the New China Digital Seismograph Network (IC; https://doi.org/10.7914/SN/IC); the IRIS/IDA Seismic Network (II; https://doi.org/10.7914/SN/II); the International Miscellaneous Stations (IM; https://www.fdsn.org/networks/detail/IM/); the Global Seismograph Network (IU; https://doi.org/10.7914/SN/IU), and the Pacific21 (PS; https://www.fdsn.org/networks/detail/PS/). The moment tensor solutions are obtained from the GCMT catalog (https://www.globalcmt.org/CMTsearch.html). The CRUST 1.0 model is available at https://igppweb.ucsd.edu/~gabi/crust1.html. The fracture zone data is obtained from the Global Seafloor Fabric and Magnetic Lineation Data Base Project website (http://www.soest.hawaii.edu/PT/GSFML/).
