# Supplementary material for: Consecutive ruptures on a complex conjugate fault system during the 2018 Gulf of Alaska earthquake
Source: Sci Rep. 2021 Mar 16;11:5979. doi: 10.1038/s41598-021-85522-w (PMC7966370; doi:10.1038/s41598-021-85522-w)
Supplement: Supplementary file 1 — Supplementary information. [file 41598_2021_85522_MOESM1_ESM.pdf]

Supporting Information for

Consecutive Ruptures on a Complex Conjugate Fault System During the 2018 Gulf of Alaska Earthquake

Shinji Yamashita[1, *], Yuji Yagi[2, **], Ryo Okuwaki[2, 3, 4], Kousuke Shimizu[1], Ryoichiro Agata[4], and Yukitoshi Fukahata[5]

[1]Graduate School of Life and Environmental Sciences, University of Tsukuba, Tsukuba, Ibaraki 305-8572, Japan
[2]Faculty of Life and Environmental Sciences, University of Tsukuba, Tsukuba, Ibaraki 305-8572, Japan
[3]Mountain Science Center, University of Tsukuba, Ibaraki 305-8572, Japan
[4]COMET, School of Earth and Environment, University of Leeds, Leeds LS2 9JT, UK
[5]Japan Agency for Marine-Earth Science and Technology, 3173-25 Showa-machi, Kanazawa-ku, Yokohama 236-0001, Japan
[6]Disaster Prevention Research Institute, Kyoto University, Uji, Kyoto 611-0011, Japan
[*]Corresponding author: Shinji Yamashita (syamashita@geol.tsukuba.ac.jp)
[**]Second corresponding author: Yuji Yagi (yagi-y@geol.tsukuba.ac.jp)

Contents of this file

Material S1 to S3
Figures S1 to S13
Tables S1 to S3

Introduction

This Supporting Information contains numerical tests for validation of the developed finite-fault inversion method (Material S1, Figs. S1 to S4, and Table S1). Sensitivity of the finite-fault inversion to assumptions of model planar depth and rupture velocity is shown in Material S2, and Figs. S5 and S6. Comparison with the conventional smoothness constraints is shown in Figs. S7 and S8. Results of a numerical test using our solution of the 2018 Gulf of Alaska earthquake as input data are shown in Fig. S9. The possibility of dummy imaging of reverberations is evaluated in Material S3 and Fig. S10. Waveform fits and full snapshots of the rupture evolution for our main result are shown in Figs. S11 and S12. Fig. S13 shows a comparison between our source model and the back-projection result obtained by Krabbenhoeft et al. (2018). Table S2 shows the near-filed velocity structure used for calculating Green's function. Table S3 provides the set of smoothness constraints adopted for the main result.

Note

Numerical experiment (S1)

We perform the numerical tests to evaluate effects of the improved smoothness constraints and the horizontal non-rectangular model plane. To generate synthetic waveforms, orthogonal three faults were assumed (Fig. S1a). Then, we assume the pure strike-slip rupture which spherically spread from a hypocenter at a depth of 30 km on the central fault, named F2, with a rupture velocity of 3.0 km/s (Fig. S1a and b). The moment rate function of input model has peaks at 9 and 23 s (Fig. S1c). We add a random Gaussian noise to the calculated Green's function, for which the standard deviation is 5% of maximum amplitude of each calculated Green's function. We also add a random Gaussian noise with zero mean and a standard deviation of 1.0 μm as background noise. We generate the synthetic waveforms at 78 stations used in the inversion of the 2018 Gulf of Alaska earthquake (Fig. 2c).

We compare results of four cases: (1) the rectangular model plane and the conventional smoothness constraints; (2) the rectangular model plane and the improved smoothness constraints; (3) the non-rectangular model plane and the conventional smoothness constraints, and (4) the non-rectangular model plane and the improved smoothness constraints.

We set the horizontal rectangular model plane with a width and length of 120 km to cover the input three faults (Fig. S2a). The depth of the model plane is set to 30 km, which corresponds to the centroid depth of input source model. The spatial knot interval is set to 10 km. For the cases (3) and (4), we design the non-rectangular model plane based on the input three faults (Fig. S2d). For all cases, the potency-rate density function at each knot is represented as a linear combination of B-spline functions over a duration of 30 s with an interval of 0.8 s and the rupture front velocity set at 7.0 km/s. We adopt the improved smoothness constraints at the cases (2) and (4) by referring to the input focal mechanism (Table S1).

In the case (1), the resultant moment rate function is smoother than the input one and has only one peak at 12 s, which is about 3 s later than the first peak of the input (Fig. S2b). The normalized L2 norm, which represents the degree of misfit between the input and the resultant moment rate function (hereinafter called "the L2 norm" for simplicity), was 0.245. The snapshots show a wider potency-rate density distribution than the input, making it difficult to identify the fault geometry and interpret the source process (Fig. S3a and b). Figure S4 shows the self-normalized potency-rate function for each basis component, obtained by taking a spatial integration of the potency-rate density function for each basis component. In the case (1), the potency-rate function of $M1$ component (Kikuchi & Kanamori, 1991), corresponding to the input slip direction, is smoother than those of other components (Fig. S4). This is because the conventional smoothness constraints work to excessively smooth out the dominant basis components.

In the case (2), the moment rate function yields two peaks at 10 and 23 s, which close to the input peaks (Fig. S2c). The L2 norm is 0.074. The improved smoothness constraints remove the bias in the resultant potency-rate function of the $M1$ component (Fig. S4) and thus the spatiotemporal potency-rate density distribution of the case (2) is

finer than that of the case (1) (Fig. S3c). However, in the case (2), the image looks too blurry to resolve two independent ruptures of the input model due to insufficient spatial resolution (snapshot at 15 and 20 s in Fig. S3c).

In the case (3), the moment rate function has two peaks at 11 and 23 s, which close to the input peaks (Fig. S2e). The L2 norm is 0.135 and slightly larger than the case (2). The potency-rate density distribution of the case (3) at 15 and 20 s resolves two ruptures, which are not well resolved in the case (2) (15 and 20 s in Fig. S3d). The spatial resolution of the inversion results is improved because the model space modification according to the input fault geometry is identical to implicitly introducing *a priori* constraint of the fault geometry (e.g., aftershock distribution). The model space reduction also contributes to reduce computational costs, which is useful for analyses of earthquakes having a vast source area, such as the 2018 Gulf of Alaska earthquake.

In the case (4), the moment rate function reproduces the input in detail (Fig. S2f), and the rupture evolution is fine enough to reproduce the input (Fig. S3e). The L2 norm of the case (4) is 0.071, which is the minimum value among the four cases. Thus, we conclude from our numerical tests that the optimum strategy should be by using both the improved smoothness constraints and the horizontal non-rectangular model plane.

Sensitivity test (S2)

We evaluate the sensitivity of the inversion results by perturbating the model parameters. We perform the inversion analyses by changing the model plane depth to 33.6$\pm$5 km. The obtained snapshots show the rupture pattern is insensitive to the model planar depths (Fig. S5). We also check the inversion results by changing the assumption of maximum rupture velocity to 3 and 5 km/s. We resolve the similar rupture processes for the maximum rupture velocities at 5 and 7 km/s (Fig. S6b and c). However, when assuming 3 km/s, the model does not clearly show the A2 rupture (Fig. S6a). This is due to the limited model space that could artificially vanish the possible slip behavior beyond the designated rupture front.

Verification of using empirical Green's functions (S3)

As shown in Figs. 2 and S11, our finite-fault model sufficiently reproduces the complicated observed teleseismic *P* waves, resulting in showing the complex-multiple rupture episodes. On the other hand, pulses of the observed waveforms may include later arrivals due to structure complexities in the source region (e.g., Fan & Shearer, 2018; Yue et al., 2017). However, the theoretical Green's functions, assuming a 1D-layered structure model, are often poorly modeled for reverberations of dipping near-source bathymetry (Wiens 1987, 1989), which may induce artificial imaging of multiple-shock sequence. In principle, seismograms of relatively smaller earthquakes with a similar focal mechanism that occurred near the target earthquake can be regarded as an empirical Green's function (EGF) under an assumption that the moment rate function of that small earthquake is simple and short (Hartzell 1978; Dreger 1994). We here employ the EGFs instead of the theoretical Green's functions to evaluate whether multiple-shock sequence that we resolve is likely from the source effect or the reverberations. We deconvolve the EGFs from the

observed waveforms of the 2018 Gulf of Alaska earthquake for each station to remove the effects of the earth response including possible reverberations.

We select three events from the GCMT catalog (Dziewonski et al., 1981; Ekström et al., 2012) as the EGFs with the clear first *P*-phase motion and high signal-to-noise ratios (Fig. S10a). The EGFs and the mainshock data are band-passed between 0.01 and 2 Hz and converted into ground velocities with a sampling interval of 0.1 s. We solve the least squares problem using the non-negative least squares algorithm of Lawson and Hanson (1974). We perform deconvolution for both a maximum source duration of 65 and 27 s to evaluate the validity of the sub-events resolved after 27 s for the mainshock (Fig. S10b, c, d, and e).

The normalized moment rate functions obtained in the maximum length of 65 s show non-negligible moment release even after 27 s (Fig. S10b, c, d and e). If the subevents after 27 s were artifacts caused by the reverberations of the initial rupture, the observed waveforms would be reproducible by convolving the moment rate function up to 27 s with the EGF. However, the synthesized waveforms obtained from the 27-s-moment-rate function fails to reproduce the several pulses of the observed waveforms, while the synthesized waveforms obtained from the 65-s-moment-rate function better fits the observed waveforms, suggesting that the subevents after 27 s should be necessary to explain the observed data (Fig. S10b, c, d and e).

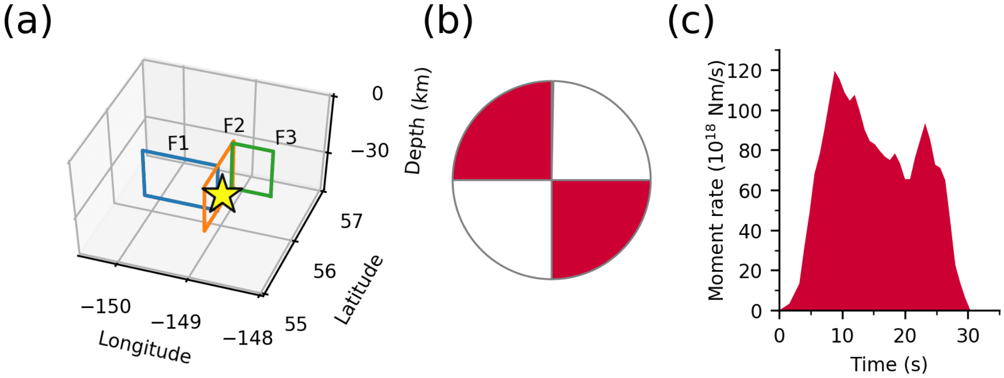

Figure S1. Input source model for the numerical tests. (a) Input fault geometry. The star indicates the location of the initial break. All three faults have dips of 90° and down-dip widths of 25 km. F1 (Blue) has a strike of 90° and a length of 65 km. F2 (Orange) has a strike of 0° and a length of 100 km. F3 (Green) has a strike of 90° and a length of 35 km. (b) Total focal mechanism of the input slip-rate. (c) Input moment rate function. This figure was made with matplotlib (v3.1.1; Hunter, 2007) and ObsPy (v1.1.0; Beyreuther et al., 2010).

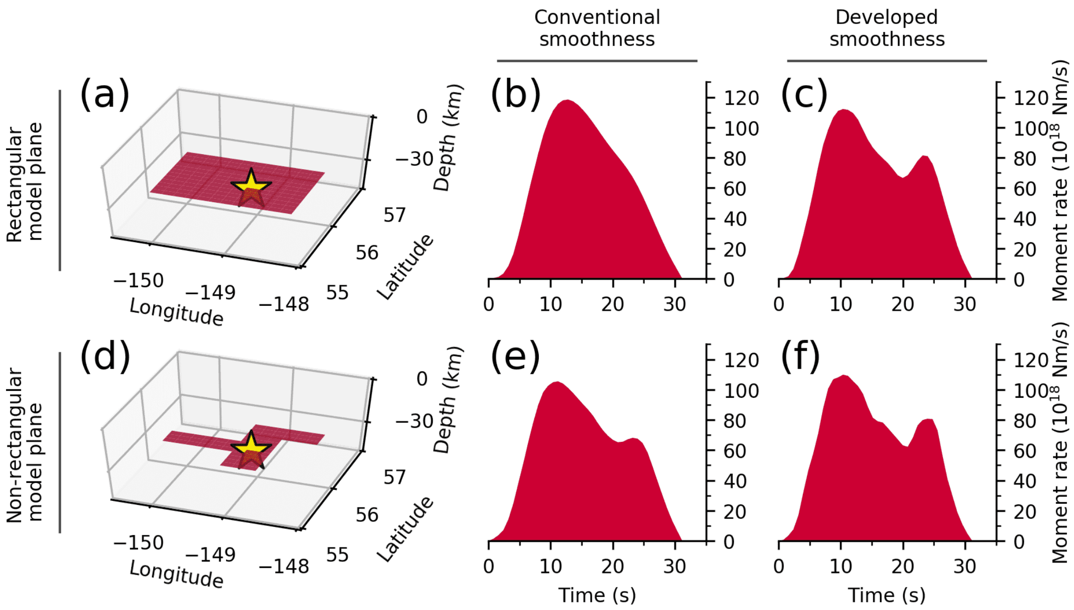

Figure S2. Assumed model planes and resultant moment rate functions for the numerical tests. (a) and (d) represent the rectangular model plane and the non-rectangular model plane, respectively. (b), (c), (e) and (f) show the moment rate functions obtained by the rectangular model plane and the conventional smoothness constraints (case 1), the rectangular model plane and the improved smoothness constraints (case 2), the non-rectangular model plane and the conventional smoothness constraints (case 3), and the non-rectangular model plane and the improved smoothness constraints (case 4), respectively. This figure was made with matplotlib (v3.1.1; Hunter, 2007).

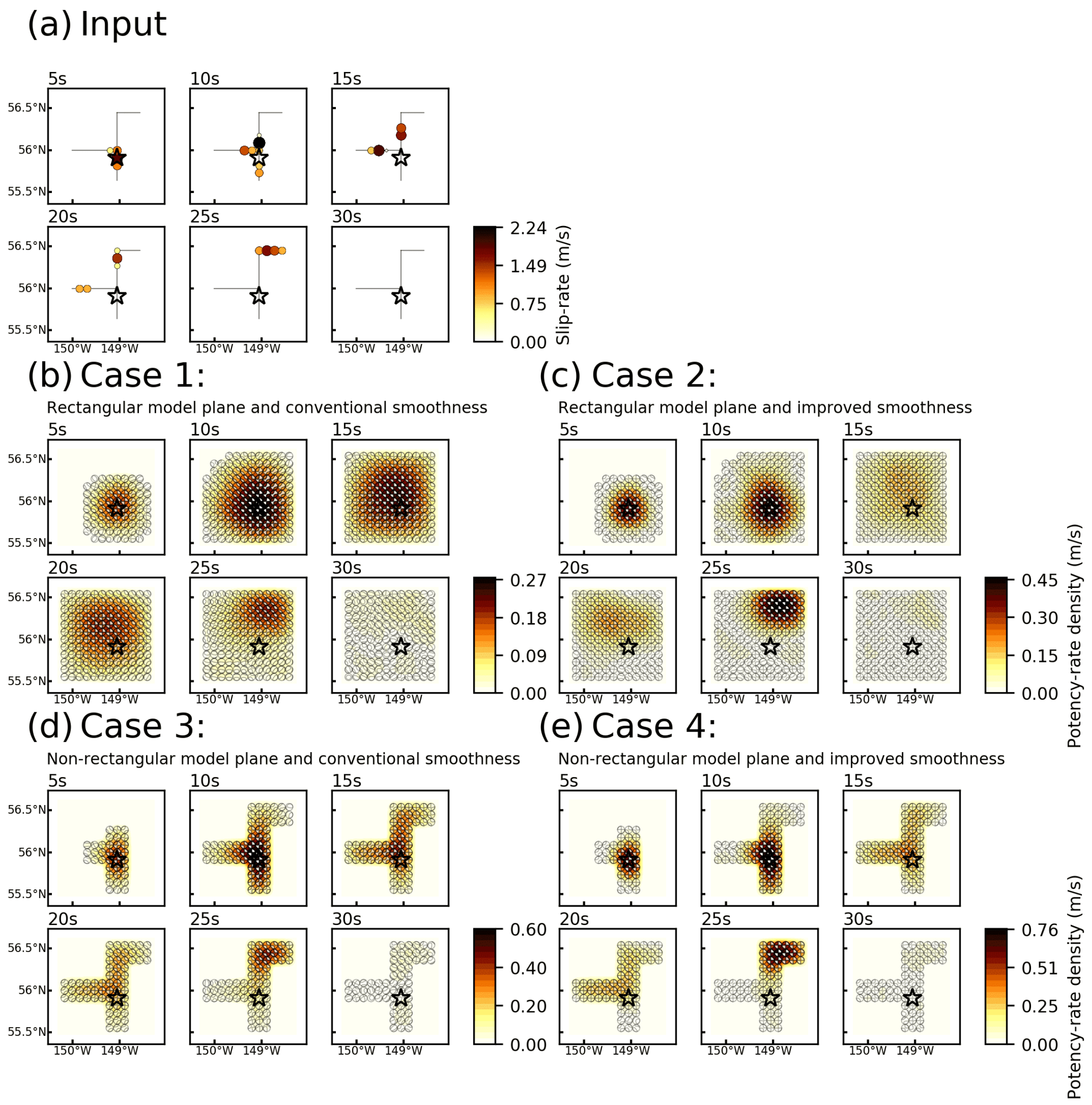

Figure S3. Snapshots of (a) input slip-rate and (b) to (e) resultant potency-rate density tensors for each numerical-test case every 5 s. The star denotes the initial breaking point. The dots in panel (a) denote the input source positions. Color of these dots represents the value of slip-rate. The gray line in panel (a) represents the input fault geometry. This figure was made with matplotlib (v3.1.1; Hunter, 2007) and ObsPy (v1.1.0; Beyreuther et al., 2010).

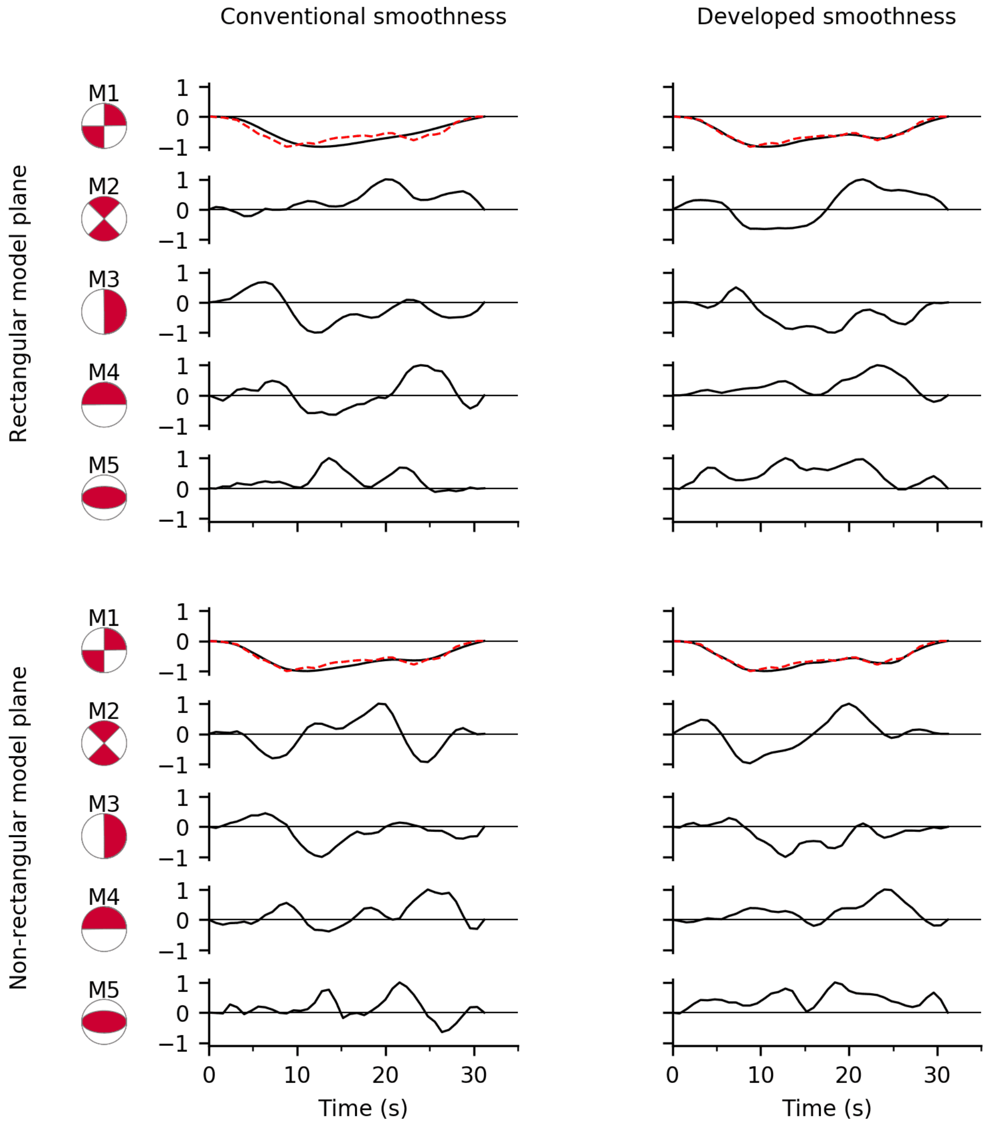

Figure S4. Comparison of the input slip-rate function (dashed line) and the potency-rate functions for each basis component, obtained by taking a spatial integration of the potency-rate density function (Kikuchi & Kanamori, 1991) (solid line). Each trace is self-normalized. This figure was made with matplotlib (v3.1.1; Hunter, 2007) and ObsPy (v1.1.0; Beyreuther et al., 2010).

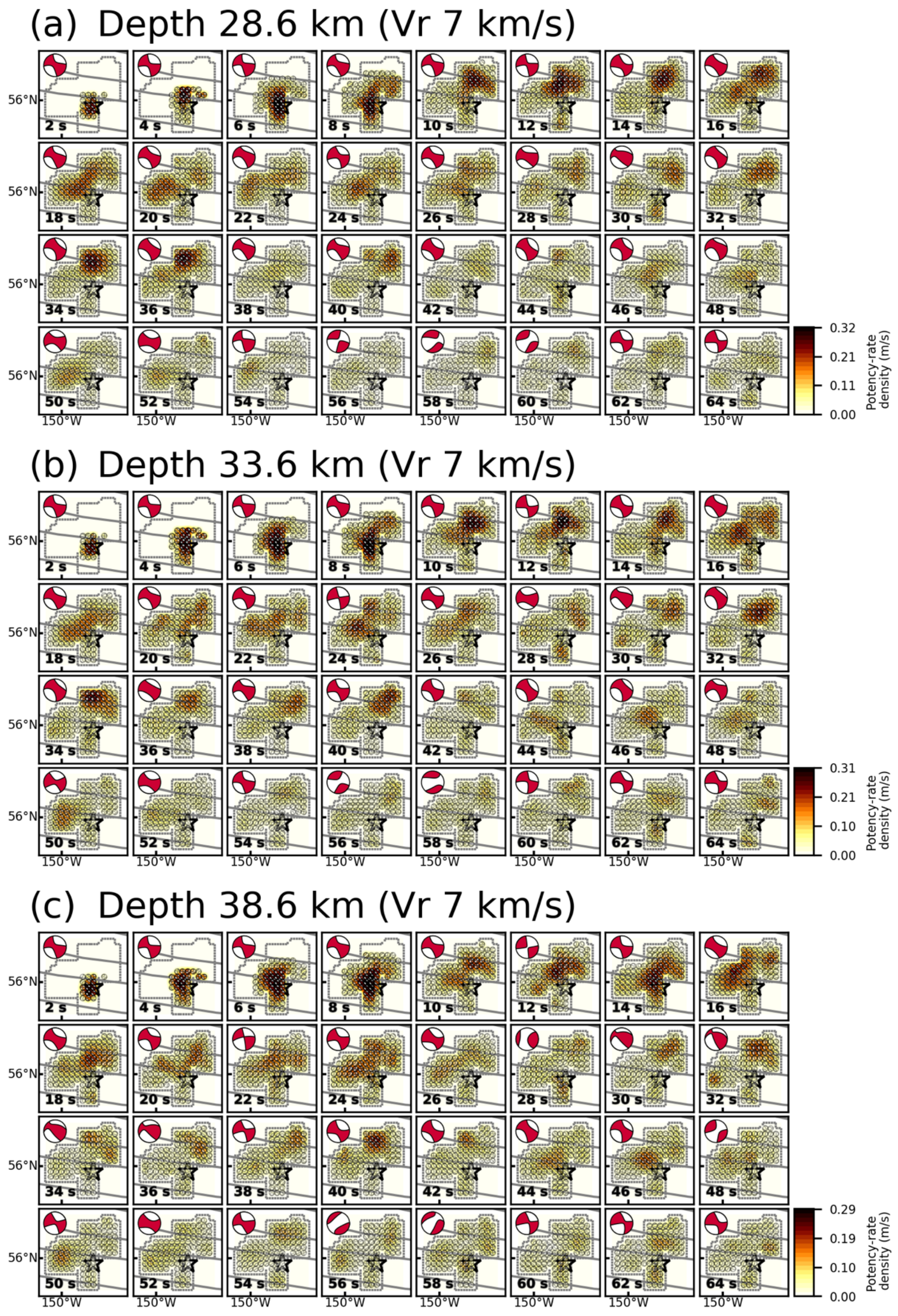

Figure S5. Summary of snapshots of the potency-rate density tensors for the different assumptions of model plane depth for the 2018 Gulf of Alaska earthquake. The depth of each snapshot is (a) 28.6 km, (b) 33.6 km, and (c) 38.6 km. The rupture front velocity (Vr) is

7 km/s for all the snapshots. The corresponding time after onset for each snapshot is noted at the bottom-left of each panel. The dotted line shows the border of the assumed model plane. The star and solid lines indicate the epicenter (AEC) and the fracture zones (Matthews et al, 2011; Wessel et al., 2015), respectively. The large beachball in each panel indicates the corresponding total moment tensor at each time. This figure was made with matplotlib (v3.1.1; Hunter, 2007) and ObsPy (v1.1.0; Beyreuther et al., 2010).

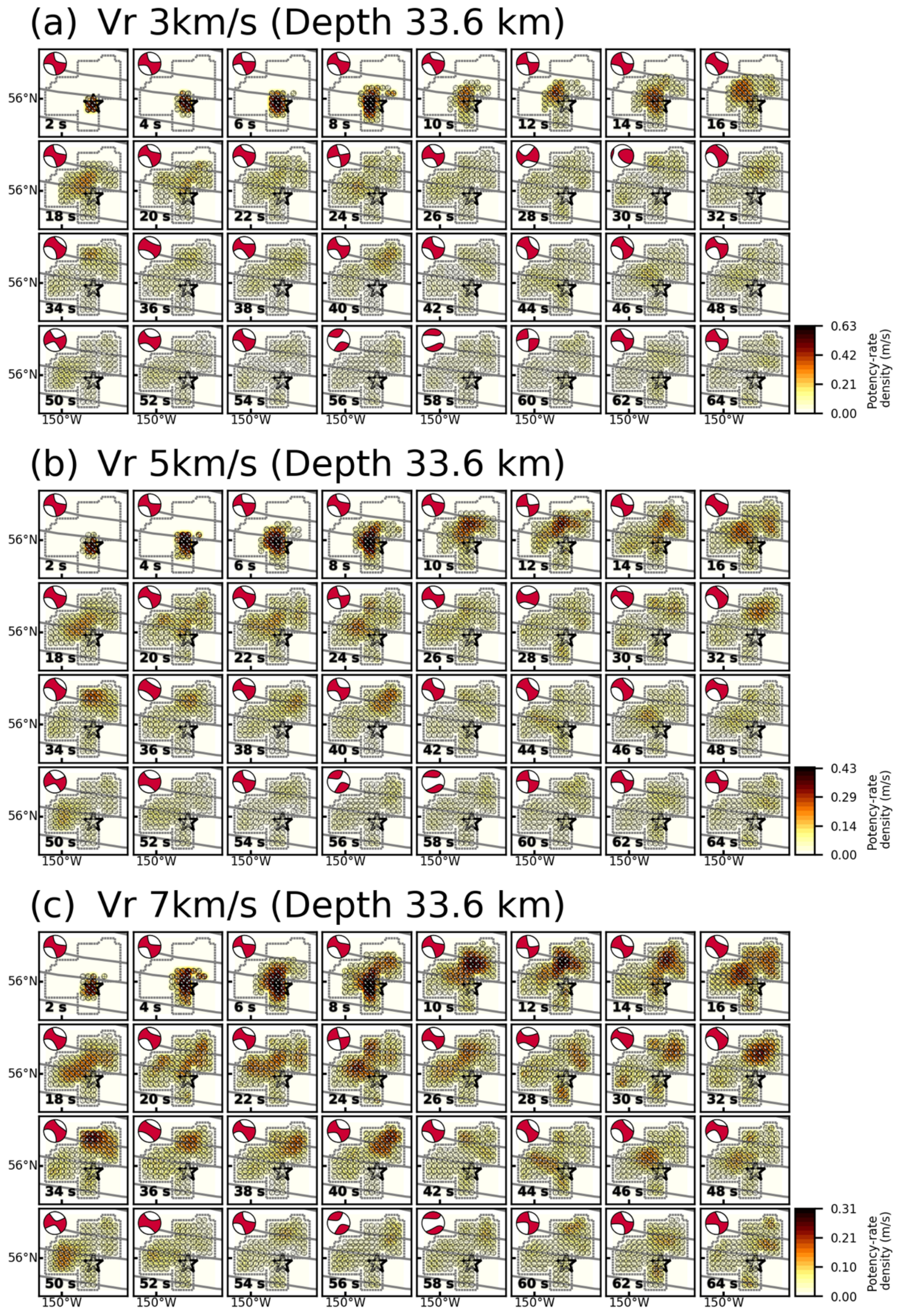

Figure S6. Summary of snapshots of the potency-rate density tensors for the different assumptions of rupture front velocity (Vr) for the 2018 Gulf of Alaska earthquake. The rupture front velocity of each snapshot is (a) 3 km/s, (b) 5 km/s, and (c) 7 km/s. The model

plane depth is 33.6 km for all the snapshots. This figure was made with matplotlib (v3.1.1; Hunter, 2007) and ObsPy (v1.1.0; Beyreuther et al., 2010).

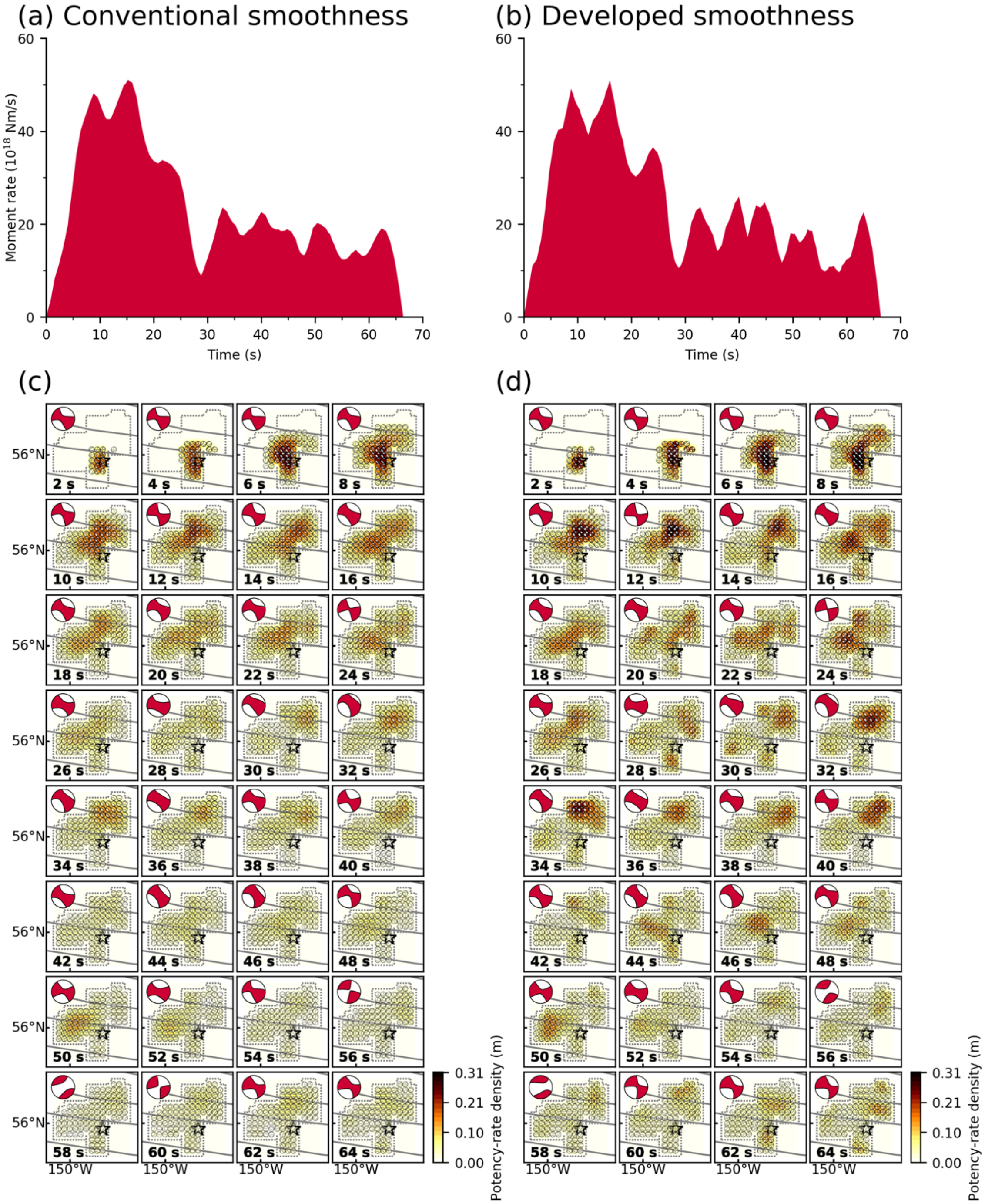

Figure S7. Comparison of results obtained by the conventional and developed smoothness constraints for the 2018 Gulf of Alaska earthquake. (a) and (b) show the moment rate functions obtained by the conventional and developed smoothness constraints, respectively. (c) and (d) show the snapshots obtained by the conventional and developed smoothness constraints, respectively. The corresponding time after onset for each snapshot is noted at the bottom-left of each panel. The dotted line shows the border

of the assumed model plane. The star and solid lines indicate the epicenter (AEC) and the fracture zones (Matthews et al, 2011; Wessel et al., 2015), respectively. The large beachball in each panel indicates the corresponding total moment tensor at each time. This figure was made with matplotlib (v3.1.1; Hunter, 2007) and ObsPy (v1.1.0; Beyreuther et al., 2010).

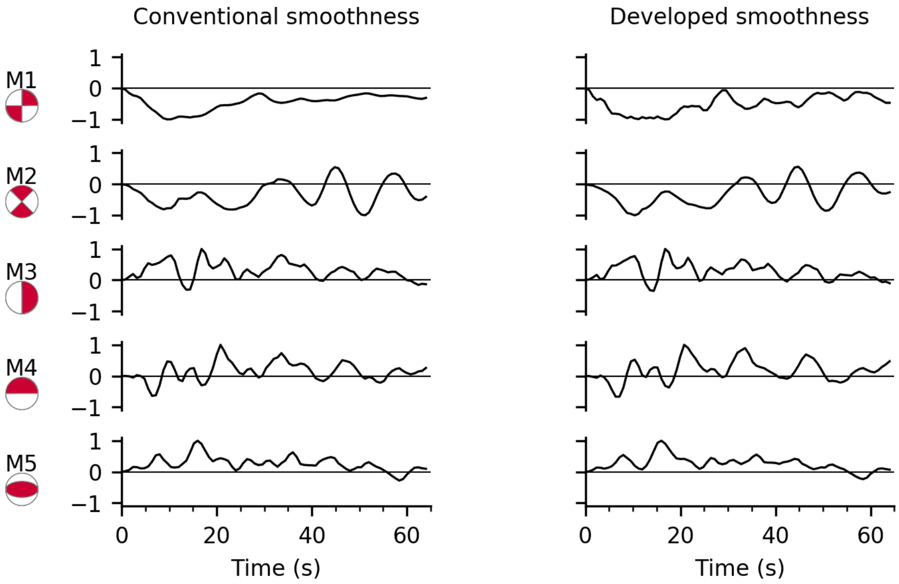

Figure S8. Comparison of the potency-rate functions of the 2018 Gulf of Alaska earthquake obtained by the conventional (left column) and improved smoothness constraints (right column) for each basis double-couple component (Kikuchi & Kanamori, 1991). Each trace is self-normalized. This figure was made with matplotlib (v3.1.1; Hunter, 2007) and ObsPy (v1.1.0; Beyreuther et al., 2010).

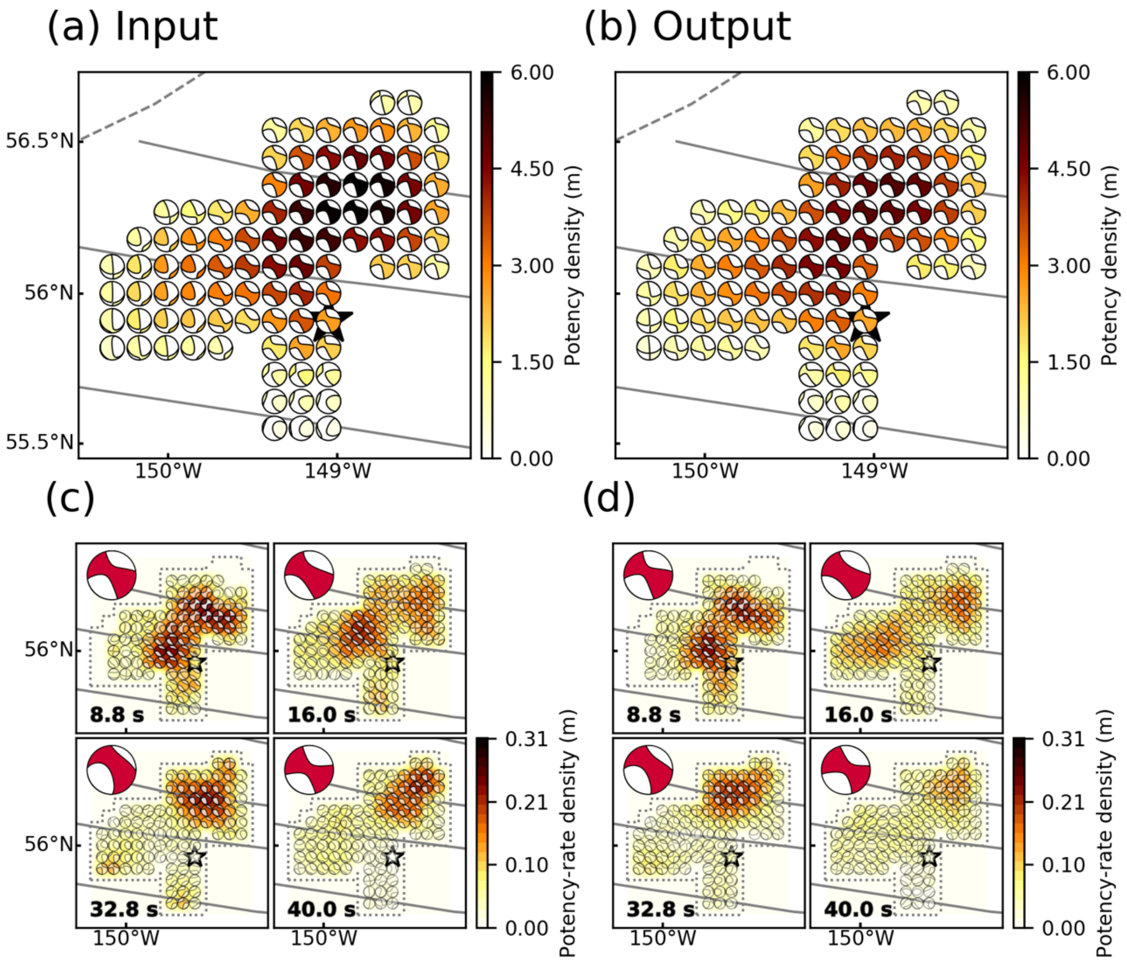

Figure S9. Sensitivity test by using the solution of the 2018 Gulf of Alaska earthquake. We inverted the synthetic waveforms of the source model (input model, Fig. 3) and evaluated the reproducibility. (a) and (b) show the potency density tensor distributions of the input and output (inversion result), respectively. (c) and (d) show the snapshots of the input and output, respectively. The hypocentral time when the snapshot is taken is on left bottom. Inset is the total moment tensor for each time. The star indicates the epicenter (AEC). The dotted line shows the border of the assumed model plane. Dashed and solid lines represent the plate boundaries (Bird, 2003) and the fracture zones (Matthews et al, 2011; Wessel et al., 2015), respectively. This figure was made with matplotlib (v3.1.1; Hunter, 2007) and ObsPy (v1.1.0; Beyreuther et al., 2010).

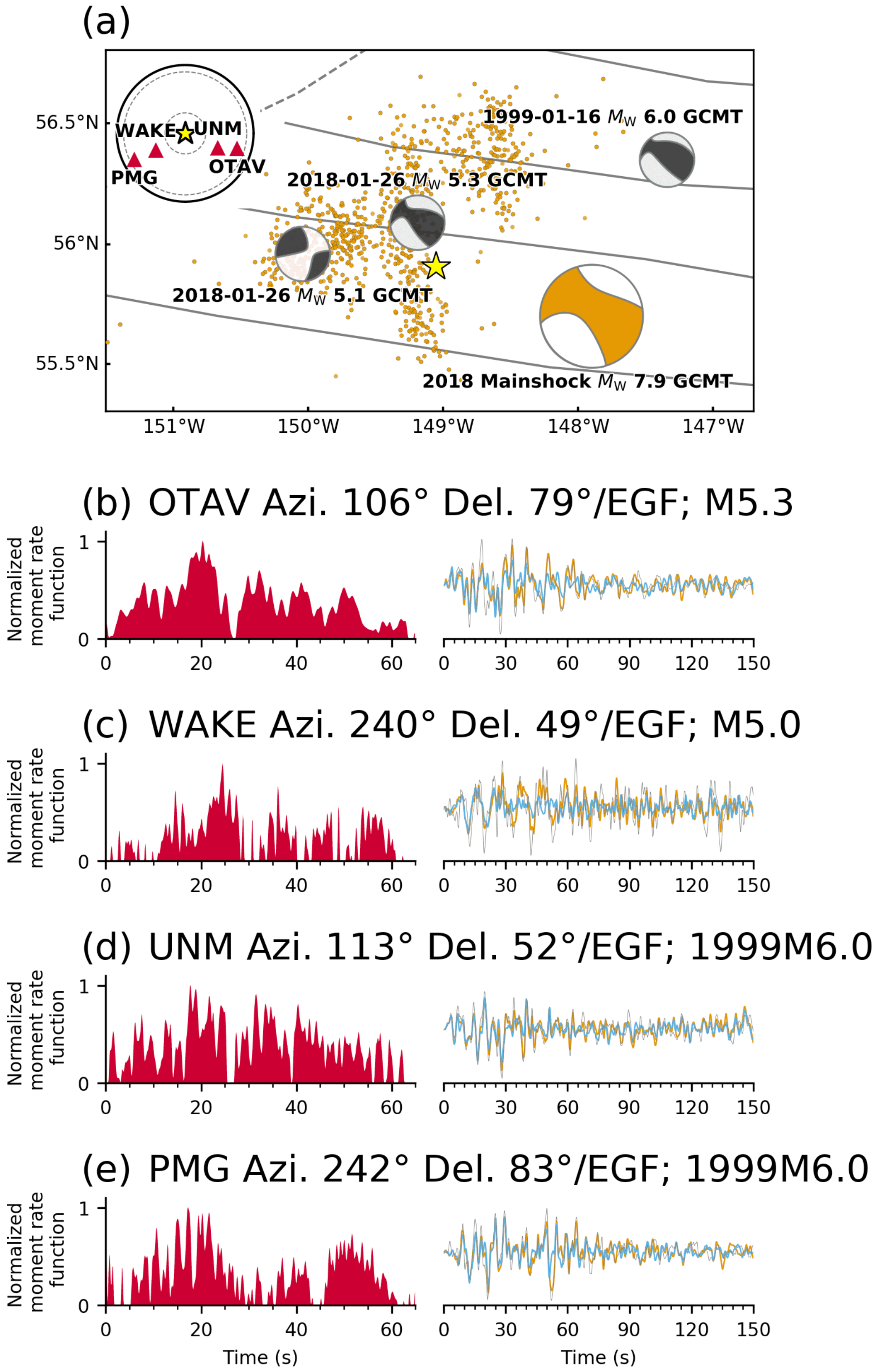

(a)
WAKE
UNM
PMG
OTAV
1999-01-16 M_W 6.0 GCMT
2018-01-26 M_W 5.3 GCMT
2018-01-26 M_W 5.1 GCMT
2018 Mainshock M_W 7.9 GCMT
56.5°N
56°N
55.5°N
151°W
150°W
149°W
148°W
147°W
(b) OTAV Azi. 106° Del. 79°/EGF; M5.3
(c) WAKE Azi. 240° Del. 49°/EGF; M5.0
(d) UNM Azi. 113° Del. 52°/EGF; 1999M6.0
(e) PMG Azi. 242° Del. 83°/EGF; 1999M6.0
Normalized moment rate function
Time (s)

Figure S10. Summary of the EGF analysis. (a) Map projection of the GCMT solutions of the main shock (orange beachball) and events used as the EGFs (black beachballs). The star is the mainshock epicenter, and orange dots are aftershocks ($M \geq 3$) that occurred within one week of the mainshock; all epicentral locations are from AEC. Dashed and solid lines represent the plate boundaries (Bird, 2003) and the fracture zones (Matthews et al, 2011; Wessel et al., 2015), respectively. Inset is an azimuthal equidistant projection of the station distribution. (b) to (e) show the normalized moment rate function (left) and waveform fittings (right). Gray trace is the observed waveform. Also shown are the synthetic waveforms obtained by using 65-s-moment-rate function (orange) and 27-s-moment-rate function (blue). Each panel is labeled with the station name, azimuth (Azi.) and epicentral distance (Del.) from the mainshock, and the event name used as the EGF. This figure was made with matplotlib (v3.1.1; Hunter, 2007) and ObsPy (v1.1.0; Beyreuther et al., 2010).

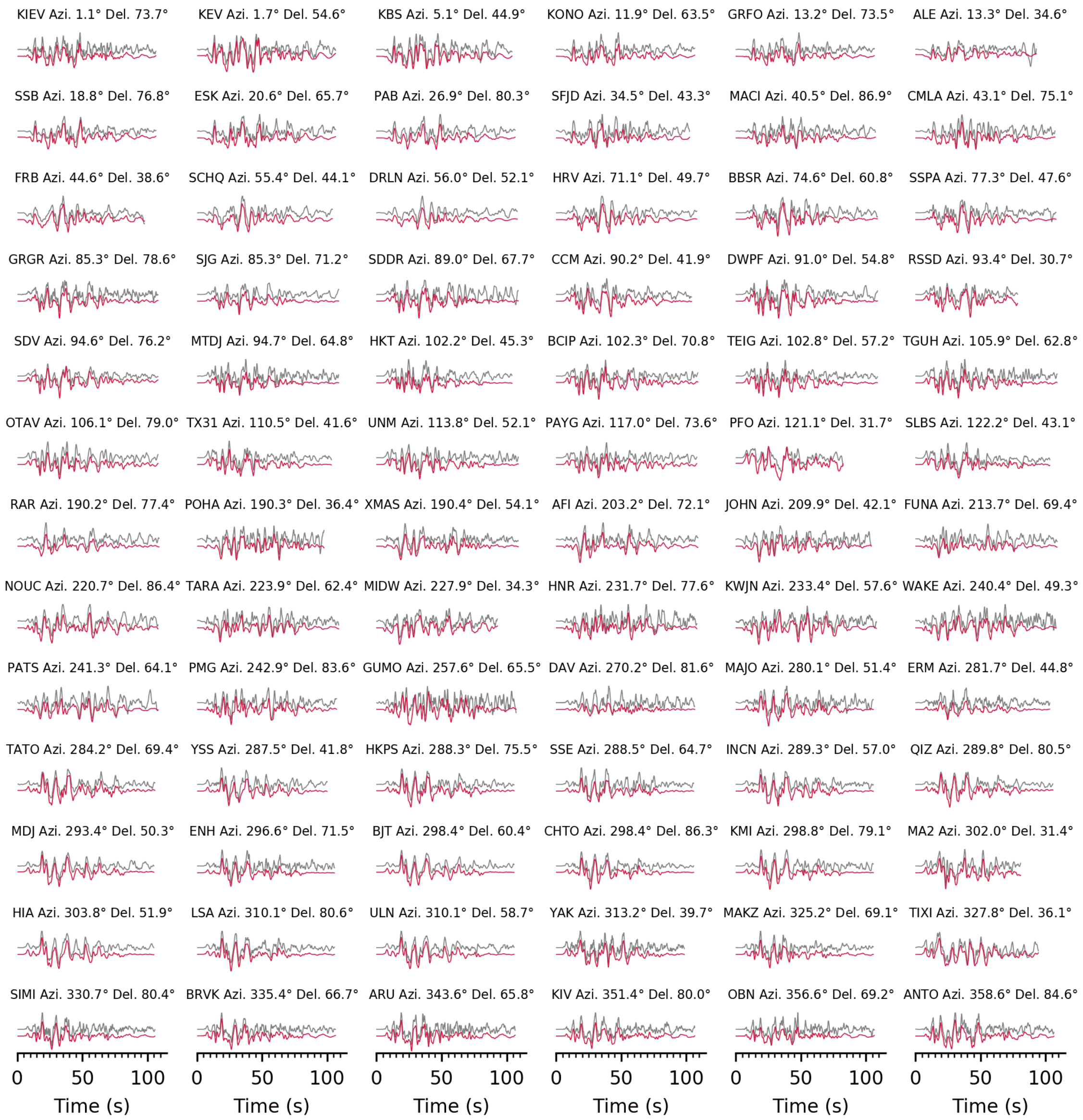

Figure S11. Comparison of observed waveforms (gray) with synthetic waveforms (red) for the main inversion results of the 2018 Gulf of Alaska earthquake. Each panel is labeled with the station name, azimuth (Azi.), and epicentral distance (Del.) from the mainshock. This figure was made with matplotlib (v3.1.1; Hunter, 2007).

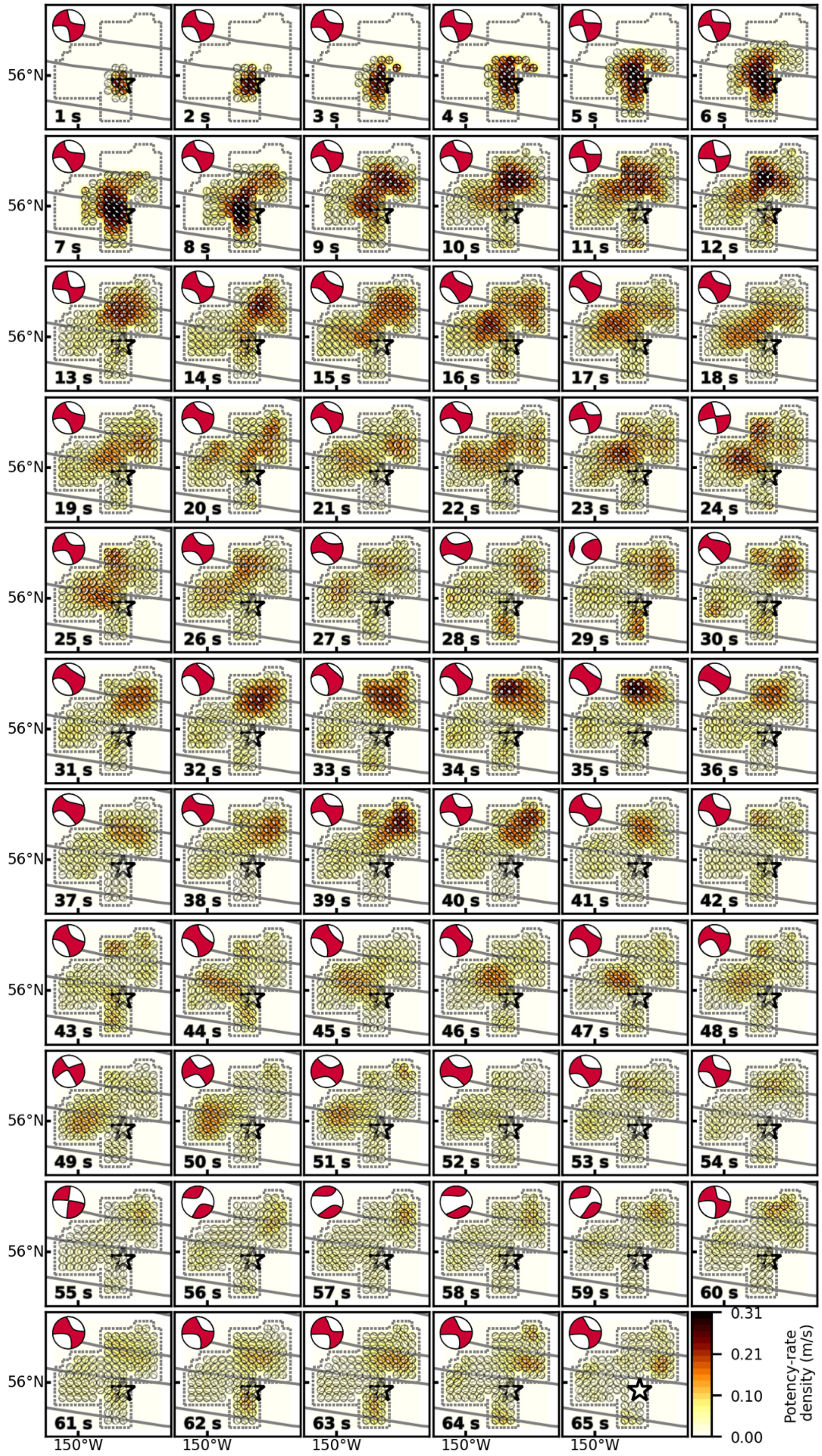

56°N
1 s
2 s
3 s
4 s
5 s
6 s
7 s
8 s
9 s
10 s
11 s
12 s
13 s
14 s
15 s
16 s
17 s
18 s
19 s
20 s
21 s
22 s
23 s
24 s
25 s
26 s
27 s
28 s
29 s
30 s
31 s
32 s
33 s
34 s
35 s
36 s
37 s
38 s
39 s
40 s
41 s
42 s
43 s
44 s
45 s
46 s
47 s
48 s
49 s
50 s
51 s
52 s
53 s
54 s
55 s
56 s
57 s
58 s
59 s
60 s
61 s
62 s
63 s
64 s
65 s
150°W
0.31
0.21
0.10
0.00
Potency-rate density (m/s)

Figure S12. Snapshots of the potency-rate density tensors every 1 s for the 2018 Gulf of Alaska earthquake. The dotted line shows the border of the assumed model plane. The star and solid lines indicate the epicenter (AEC) and the fracture zones (Matthews et al, 2011; Wessel et al., 2015), respectively. The large beachball in each panel indicates the corresponding total moment tensor at each time. This figure was made with matplotlib (v3.1.1; Hunter, 2007) and ObsPy (v1.1.0; Beyreuther et al., 2010).

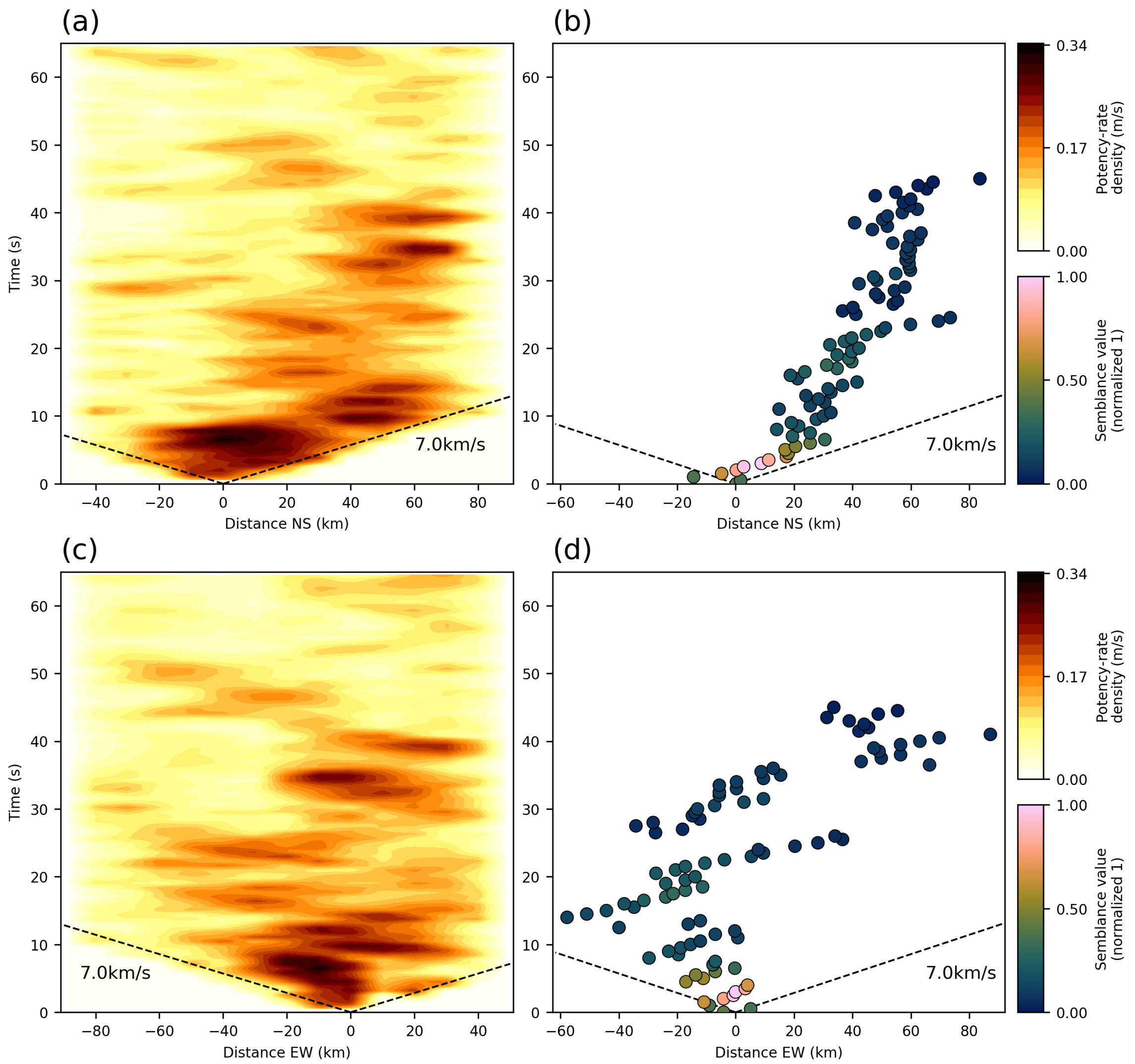

Figure S13. Comparison between the potency-rate density obtained by this study and the high-frequency (0.5-2.0 Hz) emissions obtained by Krabbenhoeft et al. (2018), projected along (a,b) north-south and (c,d) east-west directions from the epicenter. Positive distance directs toward (a,b) north and (c,d) east. Both the potency-rate density and semblance peaks distributions show general agreement in rupture pattern, especially during 0 to 10 s from the origin time. We note that a direct comparison or superimposing is not made here because our study and Krabbenhoeft et al. (2018) adopted the different reference-rupture points (epicenters). The dashed line represents the reference rupture speed. This figure was made with matplotlib (v3.1.1; Hunter, 2007).

Table S1. Factors of the smoothness constraint of each potency component for the numerical tests. The number $\boldsymbol{q}$ represents $\boldsymbol{M1}$ to $\boldsymbol{M5}$ components defined by Kikuchi and Kanamori (1991). $|\boldsymbol{m_q}|$ is the absolute value of the total potency derived from the input total moment tensor (Fig. S1b). The scaling factor $\boldsymbol{k}$ was set so that $\boldsymbol{min}(\boldsymbol{k}|\boldsymbol{m_q}|) = \mathbf{1}$.

q	1	2	3	4	5
$\|m_q\|$	1.0005	0.0000	0.0000	0.0000	0.0000
$k\,\|m_q\|$	10.0000	1.0000	1.0000	1.0000	1.0000

Table S2. CRUST1.0 structural velocity model (Laske et. al., 2013).

V_P (km/s)	V_S (km/s)	Density (10^3 kg/m^3)	Thickness (km)
1.50	0.00	1.02	4.30
1.85	0.41	1.87	0.39
5.00	2.70	2.55	0.66
6.50	3.70	2.85	1.47
7.10	4.05	3.05	4.53
8.08	4.49	3.33	0.00

Table S3. Factors of the smoothness constraint of each potency component for the analysis of the 2018 Gulf of Alaska earthquake. $|\boldsymbol{m_q}|$ is the absolute value of the total potency of each potency component derived from the GCMT solution (Fig. 1). The scaling factor $\boldsymbol{k}$ was set so that $\boldsymbol{min}(\boldsymbol{k}|\boldsymbol{m_q}|) = \mathbf{1}$.

q	1	2	3	4	5
$\|m_q\|$	0.7900	0.2500	0.3600	0.1900	0.2400
$k\,\|m_q\|$	4.1579	1.3158	1.8947	1.0000	1.2632

References

Beyreuther, M., Barsch, R., Krischer, L., Megies, T., Behr, Y., & Wassermann, J. (2010). ObsPy: A Python toolbox for seismology. *Seismological Research Letters*, 81(3), 530-533. https://doi.org/10.1785/gssrl.81.3.530

Bird, P. (2003). An updated digital model of plate boundaries. *Geochemistry, Geophysics, Geosystems*, *4*(3). https://doi.org/10.1029/2001GC000252

Dreger, D. S. (1994). Empirical Green's function study of the January 17, 1994 Northridge, California earthquake. *Geophysical research letters*, *21*(24), 2633-2636. https://doi.org/10.1029/94GL02661

Dziewonski, A. M., Chou, T. A., & Woodhouse, J. H. (1981). Determination of earthquake source parameters from waveform data for studies of global and regional seismicity. *Journal of Geophysical Research: Solid Earth*, *86*(B4), 2825-2852. https://doi.org/10.1029/JB086iB04p02825

Ekström, G., Nettles, M., & Dziewoński, A. M. (2012). The global CMT project 2004–2010: Centroid-moment tensors for 13,017 earthquakes. *Physics of the Earth and Planetary Interiors*, *200*, 1-9. https://doi.org/10.1016/j.pepi.2012.04.002

Fan, W., & Shearer, P. M. (2018). Coherent seismic arrivals in the P wave coda of the 2012 MW 7.2 Sumatra earthquake: Water reverberations or an early aftershock?. *Journal of Geophysical Research: Solid Earth*, *123*(4), 3147-3159. https://doi.org/10.1002/2018JB015573

Hartzell, S. H. (1978). Earthquake aftershocks as Green's functions. *Geophysical Research Letters*, *5*(1), 1-4. https://doi.org/10.1029/GL005i001p00001

Hunter, J. D. (2007). Matplotlib: A 2D graphics environment. *Computing In Science & Engineering*, *9*(03), 90-95. https://doi.ieeecomputersociety.org/10.1109/MCSE.2007.55

Kikuchi, M., & Kanamori, H. (1991). Inversion of complex body waves—III. *Bulletin of the Seismological Society of America*, *81*(6), 2335-2350.

Krabbenhoeft, A., von Huene, R., Miller, J. J., Lange, D., & Vera, F. (2018). Strike-slip 23 January 2018 MW 7.9 Gulf of Alaska rare intraplate earthquake: Complex rupture of a fracture zone system. *Scientific reports*, *8*(1), 1-9. https://doi.org/10.1038/s41598-018-32071-4

Laske, G., Masters, G., Ma, Z., & Pasyanos, M. (2013, April). Update on CRUST1. 0—A 1-degree global model of Earth's crust. In *Geophys. Res. Abstr* (Vol. 15, p. 2658).

Lawson, C. L., & Hanson, R. J. (1974). *Solving least squares problems,* Prentice-Hall, Englewood Cliffs., New Jersey.

Matthews, K. J., Müller, R. D., Wessel, P., & Whittaker, J. M. (2011). The tectonic fabric of the ocean basins. *Journal of Geophysical Research: Solid Earth*, *116*(B12). https://doi.org/10.1029/2011JB008413

Wessel, P., Matthews, K. J., Müller, R. D., Mazzoni, A., Whittaker, J. M., Myhill, R., & Chandler, M. T. (2015). Semiautomatic fracture zone tracking. *Geochemistry, Geophysics, Geosystems*, *16*(7), 2462-2472. https://doi.org/10.1002/2015GC005853

Wiens, D. A. (1987). Effects of near source bathymetry on teleseismic P waveforms. *Geophysical Research Letters*, *14*(7), 761-764. https://doi.org/10.1029/GL014i007p00761

Wiens, D. A. (1989). Bathymetric effects on body waveforms from shallow subduction zone earthquakes and application to seismic processes in the Kurile trench. *Journal of Geophysical Research: Solid Earth*, *94*(B3), 2955-2972. https://doi.org/10.1029/JB094iB03p02955

Yue, H., Castellanos, J. C., Yu, C., Meng, L., & Zhan, Z. (2017). Localized water reverberation phases and its impact on backprojection images. *Geophysical Research Letters*, *44*(19), 9573-9580. https://doi.org/10.1002/2017GL073254
